# Risk factors for colonization/infection by resistant microorganisms in outbreaks in neonatal unit—a systematic review and meta-analysis

**DOI:** 10.1016/j.jped.2024.12.005

**Published:** 2025-02-27

**Authors:** Roberta Maia de Castro Romanelli, Gabriela Gomes de Souza, Jordana Peruchi Fontis, José Henrique Paiva Rodrigues, João Pedro Ribeiro Viana, Kelvin Oliveira Rocha, Briana Henriques Machado Tarabai, Lêni Márcia Anchieta

**Affiliations:** a Universidade Federal de Minas Gerais, Departamento de Pediatria, Belo Horizonte, MG, Brazil; b Universidade Federal de Minas Gerais, Hospital de Clínicas, Belo Horizonte, MG, Brazil; c Universidade Federal de Minas Gerais, Belo Horizonte, MG, Brazil

**Keywords:** Newborn, Drug resistance, Antibacterial agents, Sepsis, Risk factor

## Abstract

**Objective:**

This study aims to evaluate risk factors for infection/colonization by resistant bacteria among patients in Neonatal Intensive Care Units (NICU).

**Methods:**

This systematic review is reported according to PRISMA. The search occurred by consulting the PubMed, Embase, Cochrane, SciELO, and Scopus databases. Inclusion criteria considered studies with Neonatal population admitted to the Neonatal Intensive Care Unit (P); Risk factors for resistant bacterial infection (E); No risk factors for resistant bacterial infection (C); Isolation of resistant bacteria in an outbreak (O), Observational studies (S). For Meta-Analysis, data were transformed to a logarithmic scale to directly calculate the standard error from the confidence intervals. The quality of studies was assessed Critical Appraisal Tools recommended by JBI.

**Results:**

A total of 21 articles were eligible and presented a sample size ranging from 10 to 263 newborns (a total of 1979 neonates). Six (28.6 %) studies evaluated infection, five (23.8) evaluated colonization, and 10 (47.6 %) evaluated colonization and infection, covering Gram-positive (*n* = 8; 38 %) and Gram-negative (*n* = 13; 62 %) bacteria. In the meta-analysis, the use of venous access (OR: 1.58; 95 %CI 1.14–2.20), mechanical ventilation (OR: 7.55 95 %CI 4.27–13.36), and parenteral nutrition (OR: 4.79; 95 %CI 2.23–10.29) increased the chance of colonization/infection by multiresistant microorganisms. The included studies were considered as having adequate quality.

**Conclusion:**

The main risk factors in outbreaks of infection/colonization by resistant microorganisms in Neonatal Units are the use of invasive devices and parenteral nutrition, which leads to the identification of newborns at risk, targeting the development of preventive measures.

## Introduction

Healthcare-Associated Infections (HAIs) are important conditions among the newborn population: 30 out of every 100 newborns are affected by them. In Brazil, it is estimated that 60 % of infant mortality occurs in the neonatal period, and neonatal sepsis is one of the main causes.^1^ Furthermore, there is evidence of an increase in neonatal infections caused by bacteria resistant to antimicrobials, which make these infections even more severe, with a higher mortality rate than infections caused by susceptible bacteria.^2^^,^^3^ Therefore, the relevance of studies that aim to mitigate neonatal infections caused by microorganisms resistant to antimicrobials is observed.

Although the increased incidence of infections caused by bacteria non-susceptible to antimicrobials is a challenge faced globally, newborns differ from other age groups due to their susceptibility to infections, clinical presentation, and high exposure to antimicrobials.^4^

One of the main strategies for controlling infections among the neonatal population consists of a better understanding of the risk factors and etiological agents, including the antimicrobial resistance profile. The literature describes risk factors for colonization or infection by multidrug-resistant microorganisms.^2^ However, systematic reviews may enhance the understanding of the risk factors for the neonatal infections outbreaks caused by bacteria resistant to antimicrobials, so it is possible to develop specific coping strategies against the emergence and spread of these microorganisms.

This article describes a systematic review to evaluate studies related to outbreaks of resistant bacteria among patients in Neonatal Intensive Care Units (NICU), focusing on risk factors to understand the etiology and coping strategies.

## Methods

The Preferred Reporting Items for Systematic Reviews and Meta-Analysis (PRISMA)^5^ were used to structure this systematic review, which was registered with PROSPERO (CRD42023452888). The research question was defined as: “What are the risk factors in outbreaks of infection/colonization by resistant microorganisms in Neonatal Units?”

The PECOS strategy was used, consisting of the components:

P - Neonatal population admitted to the Neonatal Intensive Care Unit
E - Risk factors for resistant bacterial infection
C - No risk factors for resistant bacterial infection
O - Isolation of resistant bacteria in an outbreak
S - Observational studies

Multidrug-Resistant Organisms are defined as bacteria resistant to one or more classes of antimicrobial agents recommended for treatment (REF: CDC https://www.cdc.gov/infection-control/hcp/mdro-management/background.html#toc).

The search for studies occurred by consulting the PubMed, Embase, Cochrane, SciELO, and Scopus databases.

As descriptors, the terms were used: “Multiple drug resistance”, “Multiple bacterial drug resistance”, “Bacterial drug resistance”, “Microbial drug resistance”, “Infant, Newborn”, “Disease outbreaks”, “Risk factors”. The search strategies are presented at Table 1.

**Table 1:** Database search strategies for “Risk factors for colonization/infection by resistant microorganisms in a neonatal unit - a systematic review”.

PubMed	((Newborn OR infant OR neonatal OR neonates) AND (NICU OR "intensive care")) AND ((Resistance OR multiresistance OR resistant) AND (Multi-drug OR multidrug OR Antibiotic OR antimicrobials OR bacteria OR bacterial OR germs OR microbe)) AND (Outbreak). The filters used were: Clinical Study, Observational Study, Newborn: birth-1 month.
EMBASE	(newborn*exp OR newborn OR 'infant'/exp OR infant OR neonatal OR neonates) AND (nicu OR 'intensive care'exo OR 'intensive care' AND ['resistance'exo OR resistance OR multiresistance OR resistent) AND (multi drug OR multidrug OR 'antibiotic'/exp OR antibiotic OR 'antimicrobials'/exp OR antimicrobials OR "bacteria'*exp OR bacteria OR bacterial OR germs OR 'microbe'/exp OR microbe AND ('outbreak'/exp OR outbreak) The filters used were: Humans, Clinical studies, Article.
SCIELO	((newborn) OR (neonatal) OR (infant)) AND ((Resistance) OR (multiresistance) OR (resistant)) AND ((Multi-drug) OR (multidrug) OR (Antibiotic) OR (antimicrobials) OR (bacteria) OR (bacterial) OR (germs) OR (microbe)) AND (Outbreak) AND ((Intensive care) OR (NICU)). No filters were used in this search.
COCHRANE	(newborn) OR (neonatal) OR (infant) in Title Abstract Keyword AND (Resistance) OR (multiresistance) OR (resistant) in Title Abstract Keyword AND outbreak in Title Abstract Keyword AND (Multi-drug) OR (multidrug) OR (Antibiotic) OR (antimicrobials) OR (bacteria) OR (bacterial) OR (germs) OR (microbe) in Title Abstract Keyword AND (Intensive care) OR (NICU) in Title Abstract Keyword - (Word variations have been searched). No filters were used in this search.
SCOPUS	(newborn OR neonates) AND (neonatal AND intensive AND care AND unity OR nicu) AND (resistance OR multiresistance OR resistant) AND (multi-drug OR multidrug OR antibiotic OR antimicrobials OR bacteria OR bacterial OR germs OR microbe) AND (outbreak) AND (LIMIT-TO (SUBJAREA, "MEDI")) AND (LIMIT-TO (EXACTKEYWORD,"Infant, Newborn")) AND (LIMIT-TO (DOCTYPE, "ar")) AND (LIMIT-TO (SRCTYPE, "j")). The filters used were Medicine, Article, Journal, Newborn.

The included studies were verified by two independent evaluators and met the following criteria: be published until June 2023; be available in any language; observe; and present a clinical observational research study.

To select publications, the title and abstract were initially evaluated to confirm whether they addressed the research question and met the previously established inclusion criteria. If necessary, the study was read in full.

As exclusion criteria, studies were removed if the neonatal population was not evaluated. Studies that did not present data necessary for extraction and analysis, or if there were duplicates were also removed.

For data extraction, a full analysis of the pre-selected studies was carried out by two independent researchers. Discrepancies were resolved by a third author. The extraction was compiled according to PRISMA,^5^ for subsequent analysis and qualitative evaluation of the studies.

For Meta-analysis, R language (4.3.3) was used. Data were transformed to a logarithmic scale to directly calculate the standard error from the confidence intervals. The evaluations were conducted using a random effects model, which uses the inverse variance method to define the weights. The Der Simonian-Laird estimator with Jackson's method was used to estimate tau^2^ values. The heterogeneity of the sample is expressed in I^2^, which is considered substantial when I2 > 50 %. Publication bias was assessed subjectively by funnel plots.

After data extraction, Critical Appraisal Tools recommended for cohorts and case-control studies by JBI^6^ scale was used to assess the quality of the articles analyzed.

## Results

**Figure 1:**
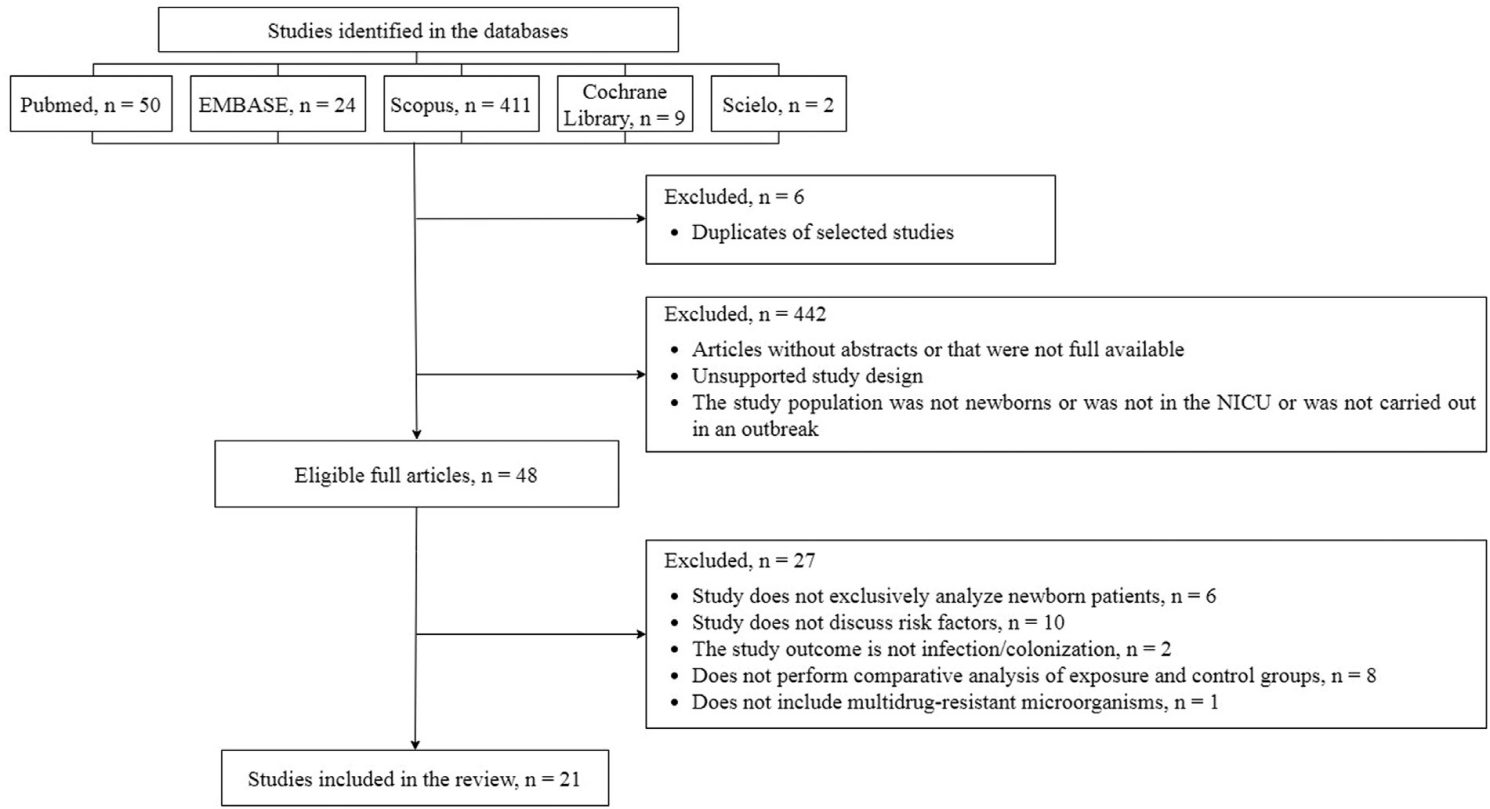
Flowchart of the Systematic Review - Assessment of Risk Factors for Outbreaks by Multiresistant Microorganisms in Neonatal Units (until 2023).

The initial search in the databases resulted in 496 studies: 411 in Scopus, 50 in PubMed, 24 in Embase, nine in the Cochrane Library, and two in SciELO. From 496 studies, 48 pre-selected studies were eligible for complete reading. According to the PECOS question, 21 articles were included in this systematic review, as presented in a flowchart in Figure 1.

There were 48 studies selected from which risk factor variables associated with outbreaks of multidrug-resistant bacteria in Neonatal Units were extracted. After complete reading, 21 articles were eligible for extraction and analysis (Table 2).

**Table 2:** Data extracted from the 21 eligible articles publish from 1980 to 2021.

First author (location, year)	Study design	Period of study	Hospitalized newborns	Outcome	Bacteria involved in the outbreak	Cases/exposed	Controls/not exposed	Significant risk factors	OR (95% IC)	P-value
Crellen et al.^26^	Cohort	09/2013-09/2014 (12 months)	333	Colonization	*Klebsiella pneumoniae (3CG-R)*	109	82	Use of Ampicillin + Gentamicin	1.96 (1.18-3.36)	_
Ulu-Kilic et al*.*^7^	Case-control	07/2014-07/2015 (12 months)	149	Bloodstream infection	*Acinetobacter baumannii (XDR-AB)*	41	108	Gestational age (weeks)	_	0.028
								Peritoneal dialysis	_	0.049
								Mechanical ventilation	_	0.017
								Umbilical catheter	2.440 (1.101-5.410)	0.013
Iosifidis et al.^24^	Case-control	06/2008-12/2008 (6 months)	389	Colonization	Vancomycin-resistant *Enterococcus faecium*	33 from 59	33 from 92	Use of second-line antibiotics (glycopeptides, meropenem, cefepime, astreonam)	_	0.02
								Hospitalization period: Month 1	_	0.01
								Month 3	_	0.03
Cantey et al.^3^	Cohort	04/2011-05/2011 (1 wk)	61	Infection/ colonization	ESBL producing *Klebsiella pneumoniae*	11	50	Gestational age (weeks)	_	0.027
								Birth weight	_	0.002
								Duration in days of use of humidified heated crib	_	0.005
								Duration in days of use of conventional crib	_	0.019
								Duration of use of umbilical venous catheter in days	_	0.014
								Duration of ventilatory support by ambient air in days	_	0.005
								Bedside surgical procedures	_	0.039
								Abdominal ultrasonography	_	0.04
								Use of surfactant	_	0.014
								Length of stay in the index patient's room	_	0.002
								Exposure in patient-days	_	0.009
Rettedal et al.^8^	Case-control	11/2008 - 04/2009 (5 months)	216	Colonization	*Klebsiella pneumoniae* (CTX-M-15 - ESBL)	44	55	Mechanical ventilation	_	_
								Use of CPAP	_	_
								Oxygen treatment	_	_
								Antibiotic treatment	5.6 (2.1 - 15.3)	0.001
								Indwelling bladder catheter	_	_
								Total parenteral nutrition	_	_
								Length of stay	_	_
								Gestational age < 37 wk	7.6 (2.8 - 20.9)	<0.001
								Gestational age < 32 wk	_	_
Guyot et al.^9^	Case-control	02/2010 - 06/2010 (4 months)	263	Infection/ colonization	ESBL producing *Klebsiella pneumoniae*	23	240	Use of Cefotaxime	_	0.04
								Use of Proton Pump Inhibitor	_	< 0.0001
										
Hosoglu et al.^10^	Case-control	11/2006 - 08/2007 (9 months)	1.622 (em 2006)	Neonatal sepsis	*Acinetobacter baumannii* (MDR)	64	128	Intubation	10.2 (4.8-21.6)	<0.001
								Re-intubation	12.8 (6.2-26.7)	<0.001
								Mechanical ventilation	7.5 (3.7-14.9)	<0.001
								Total parenteral nutrition	4.4 (1.7-11.7)	0.002
								ICU length of stay (days)	_	<0.001
								Birth weight	_	0.044
Nguyen et al.^11^	Case-control	11/2003 - 06/2004 (7 months)	Not informed	Soft tissue infection	*Staphylococcus aureus* (MRSA)	Outbreak 1: 6 Outbreak 2: 24	Outbreak 1: 5 Outbreak 2: 22	_	Outbreak 2^a^	Outbreak 2^a^
								Circumcision in the ward	U^b^ (1.7-U)	<0.01
								Use of injectable lidocaine	U (2.6-U)	<0.01
								Maternal age > 30 years	U (2.1-U)	<0.01
Brito et al.^12^	Case-control	10/2001 - 03/2002 (5 months)	33	Neonatal sepsis	*Acinetobacter baumannii* (MDR)	11	22	Birth weight: > 1500g	0.17 (0.02 - 1.03)	0.05
								Age: > 7 days	0.08 (0.00 - 1.06)	0.03
								Duration of hospitalization (≥ 7 days)	26.67 (2.41 - 692.79)	<0.001
								Antibiotic use	Indefinite	0.01
								Use of carbapenems	Indefinite	<0.001
								Use of central venous catheter	17.50 (1.42-486.05)	<0.001
								Mechanical ventilation	56.00 (4.07-1781.29)	<0.001
								Daily prevalence of patients with MDR A. baumannii infection (%)	4.31 (1.46-13.00)	0.002
Khoury et al.^13^	Case-control	10/2001-01/2002 (3 months)	28	Infection/ colonization	*Staphylococcus aureus* (MRSA)	12 infected 6 colonized	10	**Risk factors for infection:**		
								Multiple pregnancy	5.36 (1.37-20.96)	_
								Gavage feeding	10.33 (1.28-83.37)	_
								Intubation Age	5.97 (1.22-29.31)	_
								Average gestational	_	0.002
								Average birth weight	_	<0.01
								Average length of stay	_	0.003
								**Risk factors for colonization:**		
								Multiple gestation	37.5 (3.9-363.1)	_
								Mean gestational age	_	0.002
								Mean birth weight	_	<0.001
Linkin et al.^14^	Case-control	06/1998 - 12/1998 (6 months)	Not informed	Infection/ colonization (development of antimicrobial resistance)	ESBL-producing enterobacteriaceae (*Klebsiella pneumoniae e Escherichia coli)*	4	6	Estimated gestational age	_	0.03
								Duration of prior use of 3rd generation cephalosporin	_	0.02
Van der Zwet et al*.*^15^	Case-control	09/1997 - 11/1997 (2 months)	Not informed	Infection/ colonization	Gentamicin-resistant *Klebsiella pneumoniae*	8	16	None of the risk fartors analyzed were statistically significant.	_	_
Hedberg et al.^16^	Case-control	07/1987 - 10/1987 (3 months)	146	Conjunctivitis	Erythromycin-resistant *Staphylococcus aureus*	10	20	Nurse A - initial care and bathing	9	0.01
								Childbirth performed by Physician A	2.7	0.03
Balamohan et al*.*^17^	Case-control	04/2017 - 03/2018 (11 months)	536	Colonization	Staphylococcus aureus (MRSA)	50	50	Unit on the day of culture	_	−0.033
								Respiratory support (invasive and non-invasive)	_	−0.0389
								Ear test prior to MRSA colonization or control	_	0.0126
								MRSA colonization pressure (%) during the week of new colonization or control	_	<0.0001
								MRSA colonization pressure (%) during the week prior to new colonization or control	_	<0.0001
								Surface ATP rate, week of collination detection	_	0.0091
								Surface ATP rate, week prior to colonization detection	_	0.0128
Gajic et al.^18^	Case-control	05/2018 - 07/2018 (2 months)	89	Neonatal sepsis	OXA-72-producing *Acinetobacter baumannii*	13	69	Gestational age (weeks)	_	0.033
								Type of delivery:		0.018
								Vaginal	_	_
								Cesarean section	_	_
								Apgar score at 1′	_	0.018
								Apgar score at 5′	_	0.016
								Mechanical ventilation	_	0.032
								Total parenteral nutrition	_	0.03
Brown et al.^19^	Case-control	2015 (54 days)	117	Infection/ colonization	*Staphylococcus aureus* (MRSA)	8	27	Gestational age (days)	0.95/day (0.91-0.99)	0.001
								Birth weight (g)	0.997/g (0.994-0.9997)	<0.001
								Twin birth	7.30 (1.30-41)	0.02
								Nurse Exposure No. 007	7.33 (1.30-41)	0.02
								Nurse Exposure No. 033	5.75 (1.0-33)	0.049
								Nurse Exposure No. 035	15.60 (1.34-182)	0.02
								Nurse Exposure No. 045	_	<0.001
								Nurse Exposure No. 046	8.0 (1.28-50)	0.02
								Nurse Exposure No. 049	7.13 (1.17-43)	0.02
								Nurse Exposure No. 052	13.20 (2.03-86)	0.003
								Nurse Exposure No. 053	9.58 (1,61-57)	0.01
								Nurse Exposure No. 068	20.83 (2.73-159)	0.001
								Nurse Exposure No. 107	7.33 (1.30-41)	0.02
								Nurse Exposure No. 116	8.57 (1.39-53)	0.01
								Nurse Exposure No. 118	5.83 (1.07-32)	0.04
								Nurse Exposure No. 137	40.25 (3.84-421)	<0.001
								Nurse Exposure No. 148	5.75 (1.0-33)	0.048
								Nurse Exposure No. 164	16.25 (1.75-158)	0.003
								Nurse Exposure No. 178	24.50 (2.50-240)	<0.001
								Nurse Exposure No. 180	12.50 (1.69-92)	0.01
								Nurse Exposure No. 192	5.83 (1.07-32)	0.04
Andersson et al.^20^	Case-control	12/2016-05/2017 (6 months)	91	Colonization	Vancomycin-resistant *Enterococcus (VRE)*	14	77	Gestational age (WHO categories):	3.68 (1.94-7.00)	<0.001
								Extreme preterm	16.25 (3.79-62.62)	<0.001
								Gestational weight (categories):	2.68 (1.51-4.74)	0.001
								Very low weight	9.9 (1.31-74.73)	0.026
								Extreme low weight	14.14 (2.35-85.23)	0.004
								Resuscitation in Childbirth	2.37 (1.04-5.37)	0.039
								Intubation	7.1 (1.5-34.2)	0.014
								Respiratory Support:	_	_
								Ventilation	5.5 (1.45-21.24)	0.012
								CPAP	4.22 (1.28-13.99)	0.018
								High-Flow Oxygen	10.22 (1.53-68.23)	0.016
								With moisture	1.19 (1.09-1.30)	<0.001
								Total Parenteral Nutrition	5.52 (1.57-19.38	0.008
								Central venous catheter	7.44 (2.17-25.46)	0.001
								Comorbidities:	_	_
								Infection with another organism	4.92 (1.47-16.43)	0.01
								Antibiotic therapy:	_	_
								Gentamicin	4.18 (1.08-16.15	0.38
								Ampicillin	6.73 (1.20-37.61)	0.03
								Flucloxacillin	6.47 (1.79-23.43)	0.004
								Nystatin Drops	10.8 (3.05-38.30)	<0.001
								Nystatin Cream	10.8 (3.05- 38.30)	<0.001
								Antenatal Medication:	_	_
								Steroids	7 (1.85-26.46)	0.004
								Gestational weight	0.998 (0.997-0.999)	<0.001
								Period of stay	1.04 (1.02-1.06)	<0.001
								Period of CPAP use	1.04 (1.02-1.06)	<0.001
								Period of incubator use	1.12 (1.04-1.09)	<0.001
								Period of use of umblical venous catheter	1.33 (1.11-1.59)	<0.001
								Period of use of peripherally inserted central catheter	1.11 (1.03-1.20)	0.004
								Period of use of total parenteral nutrition	1.19 (1.02-1.39)	0.002
								Period of radiology use	1.15 (1.02-1.29)	0.18
Cheng et al.^21^	Case-control	09/2017 - 02/2018 (6 months)	144	Infection/ colonization	Community-associated *Staphylococcus aureus (*CA-MRSA)	15	131	Cephalosporins	49.84 (3.10-810.6)	0.006
								Duration of hospitalization, in days	1.02 (1.00-1.04)	0.013
Zarrilli et al*.*^22^	Case-control	11/2010 - 07/2011 (8 months)	161	Infection/ colonization	*Acinetobacter baumannii* (XDR)	22	139	Period of exposure to central venous catheter	5.2 (1.3-20.75)	0.019
								Use of assisted ventilation	7,01 (1,3-37.88)	0.024
Maragakis et al*.*^23^	Case-control	10/2004 - 02/2005 (4 months)	Not informed	Infection/ colonization	*Serratia marcescens* (MDR)	16	32	Presence of arterial catheter	6.33 (1.50-26.7)	0.012
								Receipt of inhalation therapy	7.22 (1.88-27.8)	0.004
Mayhall et al.^25^	Case-control	04/1977 - 06/1978 (14 months)	Not informed	Infection/ colonization	Gentamicin-resistant *Klebsiella pneumoniae (GRKP)*	18 infected 30 colonized	65	Nasopharyngeal suction	_	<0.001
								Nasogastric catheter for feeding	_	<0.001
								Ambu ventilation	_	<0.001
								Peripheral venous access	_	<0.01
								Prematurity	_	<0.01
								Umbilical Catheter	_	<0.05
								Gentamicin Therapy	_	<0.05

a Only outbreak 2 presented risk factors with statistical relevance (*P*-value < 0,05).

b U, undefined.

It was found that, among the 21 articles selected, 19 were case-controls^7^, ^8^, ^9^, ^10^, ^11^, ^12^, ^13^, ^14^, ^15^, ^16^, ^17^, ^18^, ^19^, ^20^, ^21^, ^22^, ^23^, ^24^, ^25^ and two were cohorts,^3^, ^4^, ^5^, ^6^, ^7^, ^8^, ^9^, ^10^, ^11^, ^12^, ^13^, ^14^, ^15^, ^16^, ^17^, ^18^, ^19^, ^20^, ^21^, ^22^, ^23^, ^24^, ^25^, ^26^ with the study by Crellen et al.^26^ being prospective and by Cantey et al.^3^ retrospective.

Of the 21 studies analyzed, six studies were carried out in developing countries: Turkey,^7^, ^8^, ^9^, ^10^ Brazil,^12^ Serbia,^18^ China,^21^ and Cambodia.^26^ None of the studies analyzed carried out multicenter evaluation. The other 15 studies were carried out in developed countries: Norway,^8^ France,^9^ USA,^3^^,^^11^^,^^13^^,^^14^^,^^16^^,^^17^^,^^23^^,^^25^ Netherlands,^15^ United Kingdom,^19^ Australia,^20^ Italy^22^ and Greece.^24^

The studies covered the period between 1977 and 2018. The follow-up time varied from seven days to 12 months, with the longest time observed in studies from Turkey^7^ and Cambodia.^26^

The study population corresponded to all newborns admitted to the NICU, regardless of weight or gestational age. The studied population ranged from 10 to 263 newborns, with a total of 1979 newborns. The study carried out in France was the largest in terms of population size.^9^ Regarding the number of patients hospitalized during the studies, it ranged from 28 to 536, with a total of 2756 newborns. Six studies did not report the total population in the Neonatal Unit during the period of the respective studies.^10^^,^^13^^,^^14^^,^^22^^,^^23^^,^^25^

Six studies evaluated infection,^7^^,^^10^^,^^11^^,^^12^^,^^16^^,^^20^ five evaluated colonization^8^^,^^17^^,^^20^^,^^24^^,^^26^ and ten studies evaluated colonization and infection.^3^^,^^9^^,^^13^^,^^14^^,^^15^^,^^19^^,^^21^^,^^22^^,^^23^^,^^25^

Regarding the studies that evaluated risk factors for resistant Gram-positive microorganisms, five studies evaluated an outbreak due to MRSA,^11^^,^^13^^,^^17^^,^^19^^,^^21^ and one study evaluated an outbreak due to *Staphylococcus aureus* resistant to methicillin.^17^ Two studies evaluated vancomycin-resistant *Enterococcus*.^20^^,^^24^ Regarding Gram-negative microorganisms, five studies evaluated risk factors for *Acinetobacter baumannii*, four of which defined multidrug-resistant *Acinetobacter*^7^^,^^10^^,^^12^^,^^22^ and one of them included OXA-72-producing *Acinetobacter baumannii*.^18^ Three studies evaluated Neonatal Units in which ESBL (Extended Spectrum Beta-Lactamases) producing *Klebsiella pneumoniae* was isolated,^3^^,^^11^^,^^14^ and two studies included *Klebsiella pneumoniae* resistant to gentamicin.^15^^,^^25^ Furthermore, in one study, newborns with *Klebsiella pneumoniae* resistant to third-generation cephalosporin^26^ were included. One study evaluated newborns in which ESBL-producing *Escherichia coli* was isolated^14^ and another study included newborns with isolation of multidrug-resistant *Serratia marcescens*.^23^ It is noteworthy that one of the studies included the evaluation of two microorganisms (ESBL-producing K*. pneumoniae* and *E. coli*) in the analyzed outbreak.^14^

Nineteen of the 21 assessed gestational age,^3^^,^^7^, ^8^, ^9^, ^10^, ^11^, ^12^, ^13^, ^14^, ^15^, ^16^, ^17^, ^18^, ^19^, ^20^, ^21^, ^22^, ^23^, ^24^, ^25^ 18 assessed sex^3^^,^^7^, ^8^, ^9^, ^10^^,^^12^^,^^11^, ^12^, ^13^, ^14^, ^15^, ^16^, ^17^, ^18^, ^19^, ^20^, ^21^, ^22^, ^23^, ^24^, ^25^, ^26^ and 18 assessed birth weight.^7^^,^^9^, ^10^, ^11^, ^12^, ^13^, ^14^, ^15^, ^16^, ^17^, ^18^, ^19^, ^20^, ^21^, ^22^, ^23^, ^24^, ^25^ Three studies analyzed maternal factors,^3^^,^^11^^,^^17^ two studies evaluated the use of proton pump inhibitors^3^^,^^9^ and one study evaluated the use of probiotics.^26^ Other factors analyzed were the use of: a central venous catheter,^3^^,^^7^^,^^9^^,^^11^^,^^15^^,^^17^^,^^20^^,^^21^^,^^22^ umbilical catheter,^3^^,^^7^^,^^10^^,^^15^^,^^18^^,^^22^^,^^25^ mechanical ventilation,^3^^,^^7^, ^8^, ^9^, ^10^^,^^12^^,^^15^^,^^18^^,^^20^, ^21^, ^22^^,^^24^ continuous positive airways pressure (CPAP),^3^^,^^8^^,^^9^^,^^20^ parenteral nutrition.^3^^,^^7^^,^^8^^,^^10^^,^^18^^,^^20^^,^^21^^,^^24^ Furthermore, race,^17^^,^^23^ period of hospitalization^3^^,^^10^^,^^11^^,^^13^, ^14^, ^15^^,^^22^^,^^23^^,^^24^ and type of delivery^8^^,^^11^^,^^16^^,^^18^^,^^20^ were evaluated.

Of the 19 studies that analyzed Gestational Age (GA), nine had this variable with statistical relevance, with *p* < 0,05,^3^^,^^7^, ^8^, ^9^^,^^13^^,^^14^^,^^18^, ^19^, ^20^ and the largest one demonstrated more than seven times greater chance of colonization in newborns with < 37 wk of GA.^8^

Eighteen studies analyzed the gender variable, but none achieved statistical significance. The same number of articles also analyzed birth weight and only six showed significance, associating lower weight with a higher risk of infection.^3^^,^^9^^,^^12^^,^^13^^,^^19^^,^^20^

Twelve studies analyzed mechanical ventilation as a predictor and eight had statistical significance,^3^^,^^7^^,^^8^^,^^10^^,^^12^^,^^18^^,^^20^^,^^22^ and one of them showed a more than seven times greater chance of infection in patients with mechanical ventilation.^10^ Seven articles highlighted the period of hospitalization,^3^^,^^8^^,^^10^^,^^11^^,^^12^^,^^13^^,^^21^ the largest of which demonstrated approximately 26 times greater chance of infection in newborns with >7 days of hospitalization.^12^

Among the eight articles that analyzed parenteral nutrition, two articles were able to associate its use with infection^10^^,^^18^ and two with colonization,^8^^,^^20^ with statistical significance reaching four times greater chance.^10^ Seven studies were dedicated to evaluating the use of umbilical catheters associated with infection/colonization, three obtained significant results.^3^^,^^7^^,^^25^ There were still three studies that achieved significance by associating intubation with neonatal infection/colonization,^3^^,^^7^^,^^25^ the largest one demonstrated an increased chance of infection by >10 times.^10^

Nine articles analyzed the use of central venous catheters (CVC), and three of them achieved statistical significance,^12^^,^^20^^,^^21^ the largest one presenting 56 times greater chance of infection in newborns with CVC.^12^

Regarding the use of antimicrobials, a great heterogeneity was observed. Fifteen of them assessed the use of antimicrobials as a categorized variable and a greater chance of infection/colonization was observed in nine of them.^8^^,^^9^^,^^12^^,^^14^^,^^20^^,^^21^^,^^24^^,^^25^^,^^26^ Eight studies evaluated specific classes of antimicrobials.^9^^,^^12^^,^^14^^,^^20^^,^^21^^,^^24^^,^^25^^,^^26^ Gentamicin was evaluated by Andersson et al.^20^ and by Mayhall et al.,^25^ while cephalosporins were included in studies by and Linkin et al.^14^ and by Cheng et al.^21^ The most significant study associated Cephalosporins with infection/colonization, achieving >49 times greater chance with their use.^21^ Other studies also achieved statistically significant results associating Carbapenems with a 17 times greater chance of infection/colonization.^12^ Gentamicin was associated with a six times greater chance of infection by a resistant microorganism, while nystatin had a 10 times greater chance of the same outcome occurring.^20^ A study evaluated Flucloxacillin and found a six times greater chance of colonization with its use.^20^ Two studies analyzed the use of antibiotics without class specification,^10^^,^^12^ with a significative association between ATB use and a five times greater chance of infection/colonization.^10^

Only one study^26^ considered protective factors in the analysis, however, none of them presented variables statistically significant associated with the reduction of infection/colonization by resistant bacteria.

The quality assessment of the studies was carried out according to the recommendations of the JBI Critical Appraisal Tools.^6^

Of the total of 21 studies, two had a cohort design and 19 were case-control studies. All the 21 articles were included in this systematic review. Regarding the case-control studies, all the studies received “yes” for the first, fifth, eighth, ninth, and tenth checklist items. Seven studies did not assure the second item, because it was not possible to identify any pairing method in the text.^14^^,^^18^, ^19^, ^20^, ^21^, ^22^, ^23^ Only one study did not clearly mention if the controls were defined as patients with negative bacterial cultures, which were defined as asymptomatic patients. Thus, “no” was considered for the third checklist item.^11^ The fourth item was not assured by one study, because it was not possible to find in the text objective information about the source of the patients’ data.^13^ Regarding the sixth item, seven studies did not identify any possible bias or confounding factors,^7^^,^^9^^,^^12^^,^^21^^,^^22^^,^^23^^,^^25^ but Iosifidis et al. mentioned a limitation of the study that could not clearly play the role of confounding factor. For this same reason, Iosifidis et al. received “unclear” for the seventh item. Another study also received “unclear” for this item, because, although it has described confounding factors, it was difficult to affirm the description of ways to deal with the problem.^16^ Fifteen studies did not mention any kind of strategy required in the seventh item.^7^^,^^9^, ^10^, ^11^, ^12^, ^13^, ^14^^,^^17^, ^18^, ^19^, ^20^, ^21^, ^22^, ^23^^,^^25^ In relation to cohort studies, almost all the items were fulfilled by both analyzed, except for the fact that Cantey et al. did not describe confounding factors or strategies to deal with them (fourth and fifth items) and for the tenth item, considering that there was not incomplete follow up in any of the studies. The quality evaluation is presented in Table 3.

**Table 3:** Assessment of the quality of studies using the JBI Critical Appraisal Tools recommended for cohorts and case-control studies.

Checklist case control studies												
First author (local, year)	1- Were the groups comparable other than the presence of disease in cases or the absence of disease in controls?	2- Were cases and controls matched appropriately?	3- Were the same criteria used for identification of cases and controls?	4- Was exposure measured in a standard, valid and reliable way?	5- Was exposure measured in the same way for cases and controls?	6- Were confounding factors identified?	7- Were strategies to deal with confounding factors stated?	8- Were outcomes assessed in a standard, valid and reliable way for cases and controls?	9- Was the exposure period of interest long enough to be meaningful?	10- Was appropriate statistical analysis used?	Overall appraisal	
Iosifidis et al.^24^	Yes	Yes	Yes	Yes	Yes	Unclear	Unclear	Yes	Yes	Yes	Include	
Ulu-Kilic et al*.*^7^	Yes	Yes	Yes	Yes	Yes	No	No	Yes	Yes	Yes	Include	
Rettedal et al.^8^	Yes	Yes	Yes	Yes	Yes	Yes	Yes	Yes	Yes	Yes	Include	
Guyot et al.^9^	Yes	Yes	Yes	Yes	Yes	No	No	Yes	Yes	Yes	Include	
Hosoglu et al.^10^	Yes	Yes	Yes	Yes	Yes	Yes	No	Yes	Yes	Yes	Include	
Nguyen et al.^11^	Yes	Yes	Unclear	Yes	Yes	Yes	No	Yes	Yes	Yes	Include	
Brito et al.^12^	Yes	Yes	Yes	Yes	Yes	No	No	Yes	Yes	Yes	Include	
Khoury et al.^13^	Yes	Yes	Yes	Unclear	Yes	Yes	No	Yes	Yes	Yes	Include	
Linkin et al.^14^	Yes	No	Yes	Yes	Yes	Yes	No	Yes	Yes	Yes	Include	
Van der Zwet et al*.*^15^	Yes	Yes	Yes	Yes	Yes	Yes	Yes	Yes	Yes	Yes	Include	
Hedberg et al.^16^	Yes	Yes	Yes	Yes	Yes	Yes	Unclear	Yes	Yes	Yes	Include	
Balamohan et al*.*^17^	Yes	Yes	Yes	Yes	Yes	Yes	No	Yes	Yes	Yes	Include	
Gajic et al.^18^	Yes	No	Yes	Yes	Yes	Yes	No	Yes	Yes	Yes	Include	
Andersson et al.^20^	Yes	No	Yes	Yes	Yes	Yes	No	Yes	Yes	Yes	Include	
Zarrilli et al*.*^22^	Yes	No	Yes	Yes	Yes	No	No	Yes	Yes	Yes	Include	
Mayhall et al.^25^	Yes	Yes	Yes	Yes	Yes	No	No	Yes	Yes	Yes	Include	
Brown et al.^19^	Yes	No	Yes	Yes	Yes	Yes	No	Yes	Yes	Yes	Include	
Cheng et al.^21^	Yes	No	Yes	Yes	Yes	No	No	Yes	Yes	Yes	Include	
Maragakis et al*.*^23^	Yes	No	Yes	Yes	Yes	No	No	Yes	Yes	Yes	Include	

	Checklist cohort studies											

First author (local, year)	1- Were the two groups similar and recruited from the same population?	2- Were the exposures measured similarly to assign people to both exposed and unexposed groups?	3- Was the exposure measured in a valid and reliable way?	4- Were confounding factors identified?	5- Were strategies to deal with confounding factors stated?	6- Were the groups/participants free of the outcome at the start of the study (or at the moment of exposure)?	7- Were the outcomes measured in a valid and reliable way?	8- Was the follow up time reported and sufficient to be long enough for outcomes to occur?	9- Was follow up complete, and if not, were the reasons to loss to follow up described and explored?	10- Were strategies to address incomplete follow up utilized?	11- Was appropriate statistical analysis used?	Overall appraisal
Crellen et al.^26^	Yes	Yes	Yes	Yes	Yes	Yes	Yes	Yes	Yes	No	Yes	Include
Cantey et al.^3^	Yes	Yes	Yes	No	No	Yes	Yes	Yes	Yes	No	Yes	Include

**Figure 2:**
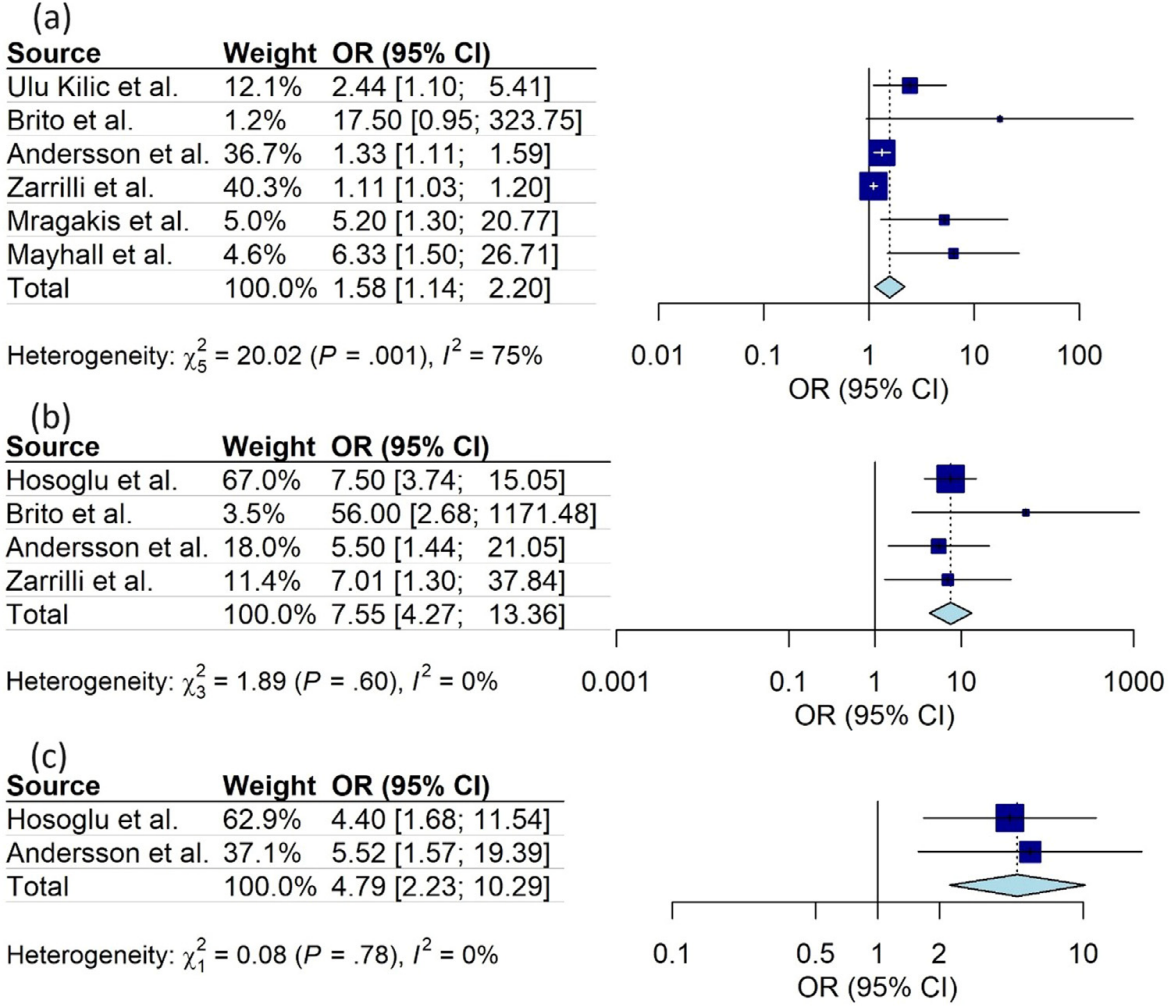
Meta-analysis for variables associated to colonization/infection by resistant microorganisms in outbreaks in Neonatal Units. (a) Use of venous access (b) Use of mechanical ventilation (c) Use of parenteral nutrition.

**Figure 3:**
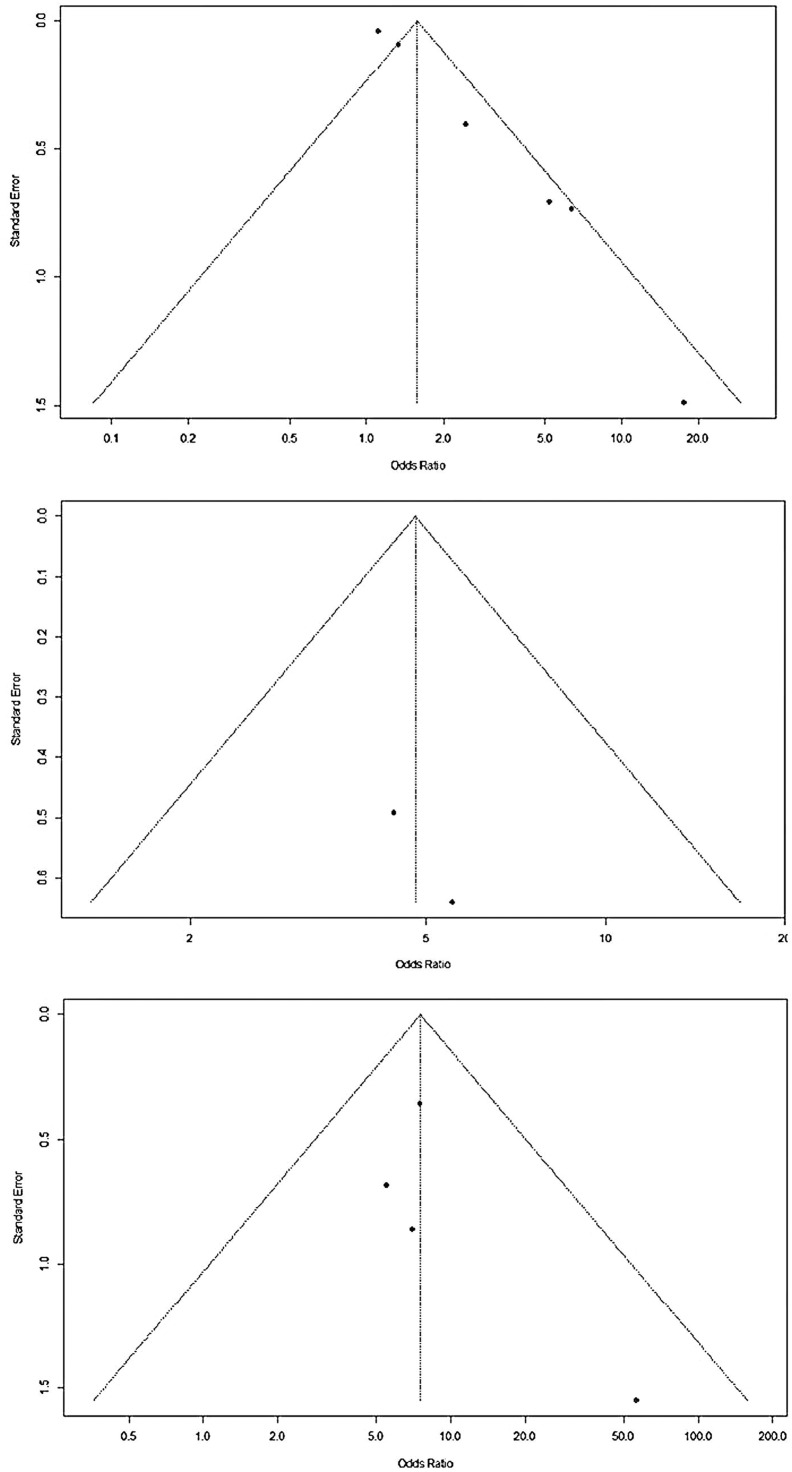
Funnel plot to access publication bias.

Meta-analysis was carried out for the same and well-defined study variables that were included in more than one study. Three variables presented a significantly higher chance of colonization or infection with multidrug-resistant bacteria: (a) use of venous access (OR 1.58; 95 %CI 1.14 - 2.20); (b) use of mechanical ventilation (OR 7.55; CI95 % 4.27 - 13.36); (c) use of parenteral nutrition (OR 4.79; CI95 % 2.23 - 10.29). The studies showed low heterogeneity in the use of mechanical ventilation and parenteral nutrition, both with I2 = 0 %. However, heterogeneity was significant regarding the use of venous access (I2 = 75 %) (Figure 2, Figure 3).

## Discussion

The main risk factors for infection/colonization by antimicrobial-resistant bacteria in NICU outbreaks were Mechanical Ventilation, Venous Access, and Parenteral Nutrition also identified in other reviews that were not focused on outbreaks.^27^^,^^28^

The temporal range of this analysis made it possible to include a greater number of patients, representing neonatal populations from different countries. It is noteworthy that over more than three decades, there have been changes in the care and structure of Neonatal Units, with a focus on reducing neonatal mortality.^29^

Early detection of outbreaks and the prompt application of preventive measures can help define research priorities and develop integrated prevention strategies for these microorganisms in the NICU.^1^^,^^30^

There was a wide variation in population size between studies, however, it is important to highlight that even the lower numbers of recorded infections/colonization by resistant microorganisms should also be treated as relevant in the neonatal population. Newborns have immunological immaturity, which favors invasive infections by these microorganisms.^31^ Therefore, identifying risk factors is relevant for the prevention and control of these infections especially when there is colonization by these pathogenic microorganisms.^29^

Colonization by resistant bacteria should also be considered as a risk factor for infection in neonates.^2^ Cantey et al.^3^ demonstrated greater lethality of infections in neonatal ICU patients infected or colonized by ESBL-producing *Klebsiella pneumoniae*, compared to patients infected by non-resistant bacteria. A study carried out in Jordan in 2017 also demonstrated a significant difference between the mortality rates of neonatal sepsis due to sepsis by resistant microorganisms compared to those with non-resistant microorganisms.^29^

Regarding the characteristics of the bacteria involved in the outbreaks reported by the selected studies, most studies included outbreaks due to Gram-negative bacteria. In developed countries, the main pathogens causing early neonatal sepsis are Gram-positive (group B Streptococcus) in full-term patients, while *E. coli*, a Gram-negative bacterium, is the most common microorganism among preterm infants with early-onset neonatal sepsis. Regarding late-onset neonatal sepsis, 15 to 30 % of cases are caused by *E.coli* or *Klebsiella species*.^2^ In very low birth weight newborns, coagulase-negative *Staphylococcus* predominates as an etiological agent of late neonatal sepsis in patients using invasive devices.^32^ Multicenter Chinese and Brazilian studies revealed that more than half of cases of late neonatal sepsis present Gram-negative bacteria as etiological agents in these countries, with emphasis on the order of *Enterobacterales*.^33^^,^^34^ Recent evidence has shown an increase in the number of neonatal infections caused by Gram-negative bacteria resistant to multiple drugs. These microorganisms are species commonly identified in neonatal sepsis, with an increasing resistance to antimicrobials. This fact demonstrates the need to optimize the use of antimicrobials in the management of neonatal infections.^2^^,^^35^^,^^36^

Approximately, one-third of the eligible studies included Gram-positive bacteria as responsible for outbreaks. The literature demonstrates that *Staphylococcus* is significantly related to late-onset neonatal sepsis and antimicrobial resistance, mainly in isolates from patients undergoing mechanical ventilation, according to extracted data from the works in this review.^13^^,^^17^^,^^35^, ^36^, ^37^

The use of broad-spectrum antibiotics favors the multiplication of resistant microorganisms and predisposes patients to colonization/infection by these agents. ESBL-producing bacteria, for example, are combated by carbapenems, a group of antimicrobials that have been identified as a risk factor for colonization/infection by bacteria with antimicrobial resistance.^12^

The use of antimicrobials was also evaluated, with emphasis on the most used to treat early neonatal sepsis (ampicillin and gentamicin) and cephalosporins, but great heterogeneity difficulted meta-analysis. Antimicrobials are essential for timely and adequate therapy for newborn infections, however, it is necessary to consider that these medications may modify microbiota, lead to adverse reactions, and develop antimicrobial resistance.^38^ Therefore, the importance of institutional programs that aim for the rational use of antibiotics in the neonatal population is necessary.^35^ Several authors have studied interventions to optimize the prescription of antimicrobials in different countries.^39^ In Sweden, demonstrated a benefit in choosing treatments of shorter duration with the support of the infectious diseases consultancy service, resulting in reduced use of meropenem-based therapy in extremely premature infants, without increasing the mortality or the need to restart treatment.^40^ In the present review, ampicillin, associated with gentamicin, was identified as a risk factor for colonization by resistant bacteria,^26^ and a study carried out in the USA demonstrated a significantly decreased use of ampicillin after the application of strategies, such as the education of multidisciplinary teams, with development of protocols on the approach to common neonatal infections.^41^ A study carried out in Brazil, demonstrated a similar result, with the application of the National Health Surveillance Agency criteria as a diagnostic tool for early neonatal sepsis reducing the number of diagnoses of this disease and the use of antimicrobials for early neonatal sepsis. There was also a reduction in general mortality and mortality related to infections after this intervention.^42^ The adoption of epidemiological surveillance systems for neonatal sepsis was identified as a contributing factor to reducing the excessive use of antibiotics in a study carried out in Spain.^32^

Although not all studies have found statistical relevance for preterm birth or low birth weight, these conditions can be associated with other situations that predispose newborns to infections, such as invasive devices (central venous catheter, umbilical catheter, mechanical ventilation) and parenteral nutrition. These devices facilitate adherence and hematogenous entry for potentially pathogenic microorganisms, predisposing newborns to HAIs.^1^^,^^29^^,^^32^^,^^43^

Protective factors against colonization/infection by multidrug-resistant bacteria were evaluated in only one of the selected studies, which did not find statistical relevance in any of the factors analyzed.^26^ However, it is noteworthy that most studies pointed to optimizing the hand washing technique of professionals in NICU as important for controlling outbreaks of multi-resistant bacteria. Horizontal transmission by hand has been described as the main source of postnatal infection in newborns admitted to hospitals.^30^ Thus, it reinforces the necessity of correct hand hygiene in the five moments recommended by the WHO before and after newborn assistance.^44^ Nguyen et al.^11^ Demonstrated that the transmission of methicillin-resistant *Staphylococcus aureus* (MRSA) was probably facilitated by inadequate hand hygiene practices. Rettedal et al.^8^ highlighted correct hand washing as the single most crucial factor in reducing the rates of nosocomial infections, besides, it is the least expensive infection control technique applied in the NICU.

The main risk factor identified as associated with multi-resistant microorganisms in outbreaks in NICU (Mechanical Ventilation, followed by Parenteral Nutrition and Venous Access), which are frequently used in NICU once these are required for assistance of preterm newborns and those with malformations, mainly those who require gastrointestinal surgery.^45^^,^^46^ For premature infants, the use of Continuous Positive Airway Pressure (CPAP) and other non-invasive ventilation used for both initial and post-intubation with timely removal of tracheal cannula may minimize the risk of lung disease and, consequently, reduce risk of infection.^47^^,^^48^ Adequacy of early and optimized Parenteral Nutrition can reduce the time of CVC use with this proposal,^49^ and bundles for the prevention of CVC-associated infections are also mandatory.^50^ The early human milk diet also reduces the time of parenteral nutrition and late-onset sepsis in newborns.^51^ Recommendations for safe surgeries and adequate preoperative prophylaxis are international policies for the prevention of infection in these patients.^45^^,^^52^

Although this review was restricted to the research question, it was directed to investigate risk factors in outbreaks, which were not identified in other studies. Several reviews included a larger number of studies that evaluated risk factors for infection in neonates despite this objective.

Thus, the best current tool for combating neonatal infections is prevention, mainly with hand hygiene practices.^35^^,^^44^ Other practices for controlling infections identified in outbreaks include the use of personal protective equipment, respiratory hygiene, patient placement and private rooms according to the transmission route, patient-care equipment and devices, and care of the environment with cleaning/disinfection.^2^^,^^53^

Despite the studies did not meet all the criteria according to the JBI Critical Appraisal Tools recommended for cohorts and case-control studies,^6^ they were included and considered as having the good quality to trust the meta-analysis results, which allows actions directed to prevent these infections.

## Conclusion

The main risk factors for infection/colonization by antimicrobial-resistant bacteria among patients admitted to NICU are the use of invasive devices such as Mechanical Ventilation, Venous Access, and Parenteral Nutrition. The best current tool is the prevention of neonatal infections, which can be achieved mainly through compliance with hand hygiene to manipulate neonates and their devices and the adoption of measures for the timely withdrawal of these interventions.

## Conflicts of interest

The authors declare no conflicts of interest.
